# Characterization of Inhibitory Capability on Hyperpolarization-Activated Cation Current Caused by Lutein (β,ε-Carotene-3,3′-Diol), a Dietary Xanthophyll Carotenoid

**DOI:** 10.3390/ijms23137186

**Published:** 2022-06-28

**Authors:** Chao-Wei Chuang, Kuo-Pin Chang, Hsin-Yen Cho, Tzu-Hsien Chuang, Meng-Cheng Yu, Chao-Liang Wu, Sheng-Nan Wu

**Affiliations:** 1 Department of Ophthalmology, Ditmanson Medical Foundation Chia-Yi Christian Hospital, Chiayi City 60002, Taiwan; 07006@cych.org.tw (C.-W.C.); 01271@cych.org.tw (K.-P.C.); 2 Department of Physiology, National Cheng Kung University Medical College, Tainan 70101, Taiwan; s36094083@gs.ncku.edu.tw (H.-Y.C.); s36091051@gs.ncku.edu.tw (T.-H.C.); s36101042@gs.ncku.edu.tw (M.-C.Y.); 3 Department of Medical Research, Ditmanson Medical Foundation Chia-Yi Christian Hospital, Chiayi 60002, Taiwan; wumolbio@mal.ncku.edu.tw; 4 Institute of Basic Medical Sciences, National Cheng Kung University Medical College, Tainan 70101, Taiwan

**Keywords:** lutein (β,ε-carotene-3,3′-diol or 3,3′-di-hydroxy-β,α-carotene), hyperpolarization-activated cation current, activation kinetics, voltage-dependent hysteresis, K^+^ current, sag potential

## Abstract

Lutein (β,ε-carotene-3,3′-diol), a xanthophyll carotenoid, is found in high concentrations in the macula of the human retina. It has been recognized to exert potential effectiveness in antioxidative and anti-inflammatory properties. However, whether and how its modifications on varying types of plasmalemmal ionic currents occur in electrically excitable cells remain incompletely answered. The current hypothesis is that lutein produces any direct adjustments on ionic currents (e.g., hyperpolarization-activated cation current, *I*_h_ [or funny current, *I*_f_]). In the present study, GH_3_-cell exposure to lutein resulted in a time-, state- and concentration-dependent reduction in *I*_h_ amplitude with an IC_50_ value of 4.1 μM. There was a hyperpolarizing shift along the voltage axis in the steady-state activation curve of *I*_h_ in the presence of this compound, despite being void of changes in the gating charge of the curve. Under continued exposure to lutein (3 μM), further addition of oxaliplatin (10 μM) or ivabradine (3 μM) could be effective at either reversing or further decreasing lutein-induced suppression of hyperpolarization-evoked *I*_h_, respectively. The voltage-dependent anti-clockwise hysteresis of *I*_h_ responding to long-lasting inverted isosceles-triangular ramp concentration-dependently became diminished by adding this compound. However, the addition of 10 μM lutein caused a mild but significant suppression in the amplitude of *erg*-mediated or A-type K^+^ currents. Under current-clamp potential recordings, the sag potential evoked by long-lasting hyperpolarizing current stimulus was reduced under cell exposure to lutein. Altogether, findings from the current observations enabled us to reflect that during cell exposure to lutein used at pharmacologically achievable concentrations, lutein-perturbed inhibition of *I*_h_ would be an ionic mechanism underlying its changes in membrane excitability.

## 1. Introduction

Lutein (xanthophyll, β,ε-carotene-3,3′-diol), derived from a hydride of a (6′R)-β,ε-carotene, is one of the few xanthophyll carotenoids that is believed to exist not only in vegetables and fruits, but also in high concentrations present in the macula of the human retina, where it is thought to act as a yellow filter [1,2,3,4,5]. Evidence from human studies suggests that dietary intake of lutein can lead to the accumulation of lutein in retinal neural tissue, thereby presumably promoting eye and brain health [6]. Moreover, the accumulation of lutein in the retina has been reported to influence the early maturation of the retina [6]. Alternatively, the beneficial bioactive effects of lutein have been widely recognized to be attributable to its antioxidant and anti-inflammatory properties. Indeed, many basic and clinical studies have growingly reported the capability of lutein to exert anti-oxidative and anti-inflammatory properties in the eye, since this compound might act as an antioxidant, protecting cells against the damaging effects of free radicals [2,4,5,6,7,8,9,10,11,12,13,14]. 

Moreover, lutein cotreatment with antiepileptic drugs was recently reported to be a promising strategy either to improve therapeutic efficacy in patients suffering from epilepsy [15] or to improve cognitive function [16]. This compound has been additionally revealed to ameliorate either ischemia-reperfusion-induced vasculitic neuropathic pain and neuropathy in rats, or photoreceptor degeneration in a mouse model of retinitis pigmentosa [17,18,19]. It was also recently reported to exert anti-neoplastic action [20,21]. Changes in oxidative stress or the activity of NADPH oxidase have been recently demonstrated to affect different types of plasmalemmal ionic currents [22]. However, to our surprise, the ionic mechanism of lutein-induced actions through which it affects the functional activities of excitable cells remains largely unanswered.

The hyperpolarization-activated cation current (*I*_h_ or funny current [*I*_f_]) is well-recognized as being a key determinant of repetitive electrical activity in heart cells and in various types of neurons and neuroendocrine or endocrine cells [23,24,25,26,27,28,29,30,31,32,33,34,35,36,37,38]. This type of ionic current comprises a mixed inward Na^+^/K^+^ current (i.e., unusual ion selectivity in that it can conduct both Na^+^ and K^+^ ions), which is vulnerable to inhibition by CsCl, ivabradine, or ZD7288 [23,26,39,40,41,42,43]. It is believed that the activation of this current, which proceeds at resting membrane potential, is allowed to produce a net inwardly directed current carried largely by Na^+^ ions, thereby bringing about membrane depolarization to the threshold critically involved in the generation of action potentials [23,25,32,44]. These currents are assumed to be carried by channels of the hyperpolarization-activated cyclic nucleotide-gated (HCN) gene family, which belongs to the superfamily of voltage-gated K^+^ (K_V_) channels and cyclic nucleotide-gated (CNG) channels [26,28,34,37,44,45,46]. HCN2, HCN3, or mixed HCN2 + HCN3 channels were reported previously to be distributed in pituitary tumor (GH_3_) cells [27,28,39]. The activities of CNG and HCN channels have also been thought to be closely linked to phototransduction intrinsically found in photosensitive retinal ganglion cells [47]. At present, the issue of whether and how lutein can interact with the HCN channel to modify the amplitude, gating properties, and hysteretic magnitude of *I*_h_ remains unsolved.

In view of the foregoing considerations, the possible electrophysiological actions of lutein and other related compounds in pituitary GH_3_ cells were explored in this study. We tried to study the effects of lutein on the amplitude and gating of *I*_h_ residing in pituitary GH_3_ somatolactotrophs. Findings from the current observations tempt us to reflect that the *I*_h_ present in different cell types could be an unidentified but distinctive target through which the lutein molecule can act to affect the functional activities of the cells involved.

## 2. Results

### 2.1. Suppressive Effect of Lutein on Hyperpolarization-Activated Cation Current (I_h_) Measured from Pituitary Tumor (GH_3_) Cells

**Figure 1:**
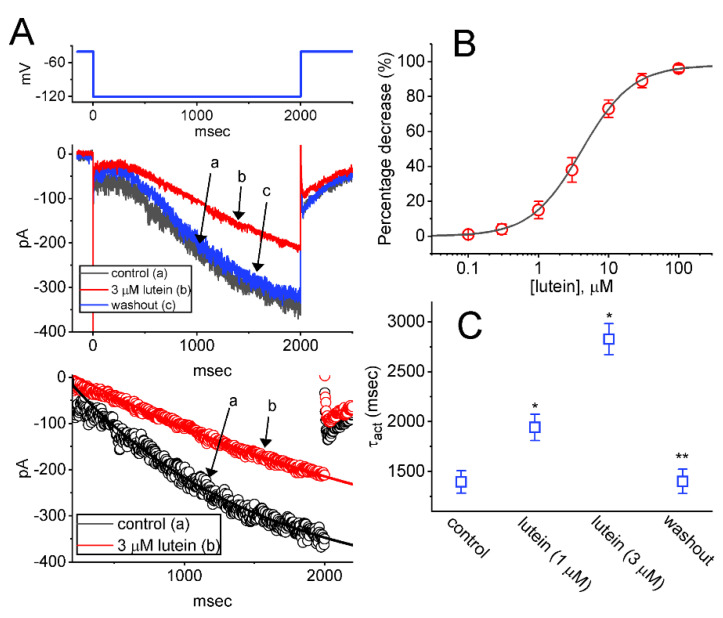
Inhibitory effect of lutein on hyperpolarization-activated cation current (*I*_h_) identified from pituitary GH_3_ cells. In this stage of experiments, we placed cells to be immersed in Ca^2+^-free, Tyrode’s solution, which contained 1 μM tetrodotoxin (TTX) and 0.5 mM CdCl_2_, and we then filled up the recording pipette by using a K^+^-containing solution (145 mM). (**A**) Superimposed current traces were obtained in the control period (a, absence of lutein, black color), during cell exposure to 3 μM lutein (b, red color), and after washout of lutein (c, blue color). The voltage protocol (i.e., 2-sec membrane hyperpolarization from −40 to −120 mV, blue color) used to evoke *I*_h_ is pointed out in the upper part. The lowest part in (**A**) demonstrates the best fit of digitized data points acquired in the absence (a, open black circles) or presence (b, open red circles) of 3 μM lutein to single exponential (indicated in smooth continuous black or red line) with a time constant of 1395 or 2927 msec, respectively. (**B**) Concentration-dependent inhibitory effect of lutein on the amplitude of *I*_h_ (mean ± SEM; *n* = 7 for each point). We measured current amplitude at the end of hyperpolarizing step, and the smooth gray line drawn was reliably fitted to a modified Hill function as described in Section 4. (**C**) Summary scatter graph showing the effect of lutein (1 or 3 μM) on the activation time constant (τ_act_) of *I*_h_ (mean ± SEM; *n* = 7 for each point). The slowly activating *I*_h_ was evoked by 2 sec hyperpolarizing step from −40 to −120 mV and the *I*_h_’s τ_act_ value was optimally estimated with single exponential. * Significantly different from control (*p* < 0.05) and ** significantly different from the lutein-alone (1 μM) group (*p* < 0.05).

In this study, we began to exploit the whole-cell configuration of the patch-clamp technique to evaluate any possible modifications of lutein on ionic currents identified in GH_3_ cells. In order to record ionic current flowing through *I*_h_, we kept cells in Ca^2+^-free, Tyrode’s solution containing 0.5 mM CdCl_2_ and 1 μM tetrodotoxin (TTX), and the recording pipette that was used was filled up with a K^+^-containing (145 mM). TTX or CdCl_2_ was used to block voltage-gated Na^+^ or Ca^2+^ currents, respectively. As the whole-cell mode was firmly achieved, we maintained the tested cell at a holding potential of −40 mV and a 2-sec hyperpolarizing step to −120 mV was thereafter imposed to evoke *I*_h_. Of interest, one minute after cell exposure to lutein, the amplitude of *I*_h_ evoked in response to such 2-sec long step hyperpolarization from −40 to −120 mV became progressively lessened (Figure 1A). For example, at the level of −120 mV, the application of the compound at a concentration of 3 μM resulted in a decrease of *I*_h_ amplitude from 338 ± 27 to 201 ± 18 pA (*n* = 7, *p* < 0.05). After the lutein was removed, the current amplitude was returned to 332 ± 25 pA (*n* = 7). Concurrently, under GH_3_-cell exposure to 3 μM lutein, the value of activation time constant (τ_act_) significantly arose from 1.4 ± 0.1 s to 2.8 ± 0.2 s (*n* = 7, *p* < 0.05).

The relationship between the concentration of lutein and the amplitude of *I*_h_ was further collated, and the results are hence presented in Figure 1B. In this stage of measurements, we held the examined cell in a voltage clamp at −40 mV, and the hyperpolarizing pulse (2-sec duration) to −120 mV was thereafter imposed on it, while current amplitudes during cell exposure to varying lutein concentrations (0.1–100 μM) were measured at the end pulse of long-lasting hyperpolarizing command voltage. It became clear that cell exposure to lutein is able to suppress *I*_h_ in a concentration-dependent manner in these cells. By virtue of a sigmoidal least-square fit to the experimental data, the value of effective IC_50_ (i.e., the concentration required for a half-maximal inhibition) for the inhibitory effect of lutein on *I*_h_ was properly yielded at 4.1 μM. Additionally, the existence of lutein increased the τ_act_ value of *I*_h_ evoked by long-lasting membrane hyperpolarization (Figure 1C). The results tempt us to reflect that, under our experimental conditions, lutein can bring about a depressant action of *I*_h_ in these cells in a time- and concentration-dependent manner.

### 2.2. Effect of Lutein on the Quasi-Steady-State Activation Curve of I_h_ Identified in GH_3_ Cells

**Figure 2:**
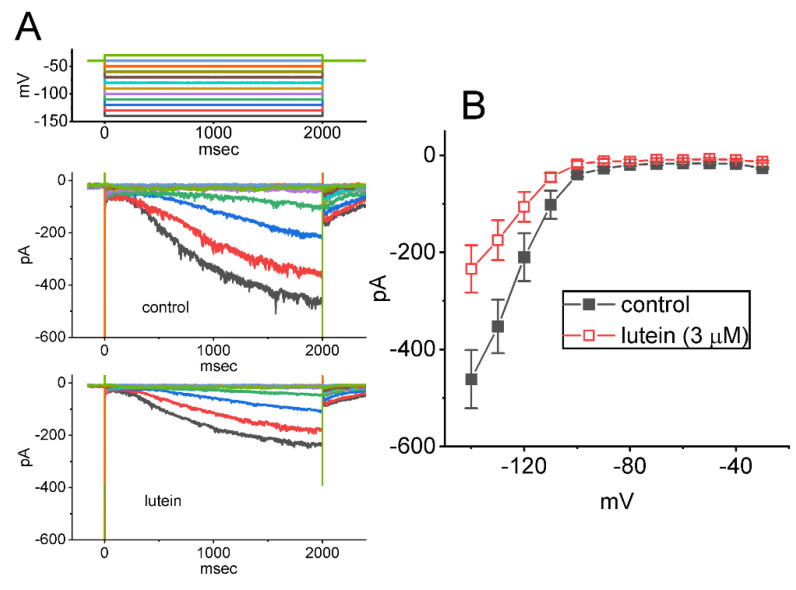

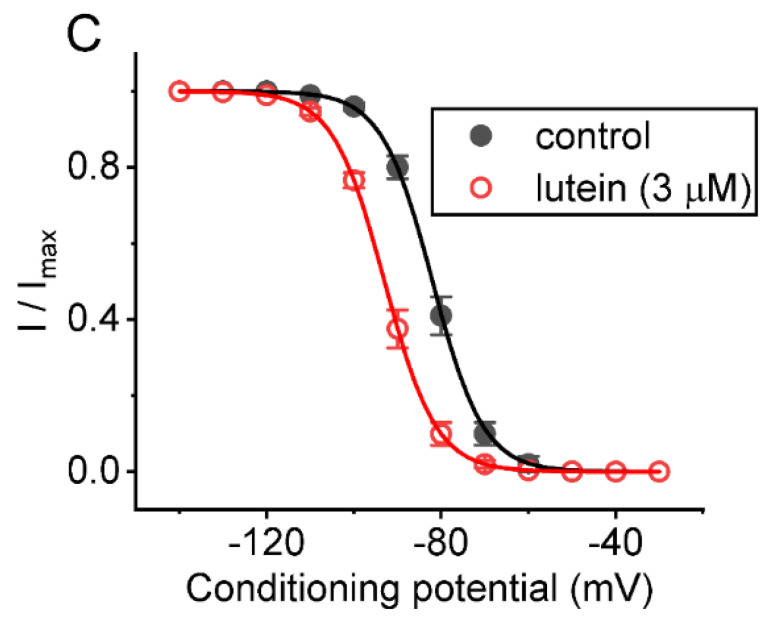
Inhibitory effect of lutein on average current versus voltage *(I*–*V*) relationship (**A**,**B**) and steady-state activation curve (**C**) of *I*_h_ recorded from GH_3_ cells. In this set of measurements, experimental protocols are the same as those appearing in Figure 1. (**A**) Representative current traces acquired in the absence (upper) and presence of 3 μM lutein (lower). The uppermost part points to the voltage-clamp profile imposed over the tested cell. Potential traces shown in different colors correspond with ionic currents that were evoked by the same level of step voltages. (**B**) Average *I–V* relationship of *I*_h_ acquired in the absence (■) or presence (□) of 3 μM lutein (mean ± SEM; *n* = 7 for each point). (**C**) Modifications of lutein (3 μM) on the quasi-steady-state activation curve of *I*_h_ seen in GH_3_ cells. Continuous sigmoidal curves drawn were optimized with the chi-square test of goodness of fit according to a modified Boltzmann function as detailed in the Section 4. Each data point is the mean ± SEM (*n* = 7). ●: control; ○: in the presence of 3 μM lutein. Of note is the leftward shift along the voltage axis in the steady-state activation curve of *I*_h_ during cell exposure to lutein.

To characterize the inhibitory effect of lutein on *I*_h_, an effort was made to ascertain if cell exposure to lutein might modify any changes in the steady-state activation curve of *I*_h_ intrinsically in GH_3_ cells. Figure 2C illustrates the steady-state activation curve of *I*_h_ acquired without or with the presence of this compound at a concentration of 3 μM. The Boltzmann distribution (or Fermi-Dirac distribution) detailed in Section 4 was applied to fit the experimental data points appropriately. In the control (i.e., in the absence of lutein), V_1/2_ = −82 ± 8 mV and *q* = 4.35 ± 0.07 *e* (*n* = 7), and during the exposure to 3 μM lutein, V_1/2_ = −93 ± 9 mV and *q* = 4.29 ± 0.08 *e* (*n* = 7). Consequently, the emerging data led us to project that during GH_3_-cell exposure to lutein (3 μM), the steady-state activation curve of *I*_h_ elicited under different preceding levels of conditioning step-pulse potential was shifted along the voltage axis toward more hyperpolarized potential (approximately 11 mV), despite no concurrent changes in the apparent gating charge (i.e., *q*) of the curve.

### 2.3. Comparisons among the Effect of Lutein, Lutein Plus Oxaliplatin and Lutein Plus Ivabradine on I_h_ Amplitude

**Figure 3:**
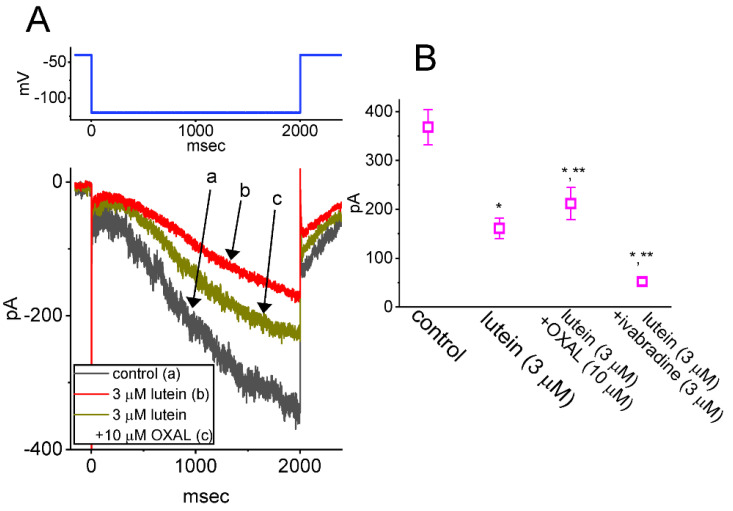
Comparisons among effects of lutein, lutein plus oxaliplatin, and lutein plus ivabradine on *I*_h_ amplitude recorded from GH_3_ cells. In these experiments, whole-cell current recordings were performed in cells immersed in Ca^2+^-free, Tyrode’s solution containing 1 μM TTX, and during the measurements, we filled up the recording pipette with K^+^-enriched internals solution. (**A**) Superimposed current traces acquired in the control period (a, black color) and during cell exposure to 3 μM lutein-alone (b, red color) or to 3 μM lutein plus 10 μM oxaliplatin (OXAL) (c, brown color). The upper part denotes the voltage protocol (blue color) imposed on the tested cell. (**B**) Summary scatter graph demonstrating the effect of lutein, lutein plus oxaliplatin (OXAL), and lutein plus ivabradine on *I*_h_ amplitude (mean ± SEM; *n* = 7 for each point). We measured each current amplitude (acquired during cell exposure to different tested compounds) at the end-point of the 2-sec hyperpolarizing step from −40 to −120 mV. In the experiments on lutein plus OXAL or lutein or ivabradine, lutein was first added to the bath before OXAL or ivabradine was further applied. * Significantly different from control (*p* < 0.05) and **significantly different from the lutein-alone (3 μM) group (*p* < 0.05).

We then investigated if the addition of oxaliplatin or ivabradine, but still in the continued exposure to 3 μM lutein, was able to adjust lutein-perturbed inhibition of *I*_h_ identified in GH_3_ cells. Oxaliplatin or ivabradine has been recently demonstrated to activate or suppress *I*_h_, respectively [40,48,49]. In this set of current recordings, we placed cells in Ca^2+^-free, Tyrode’s solution containing 1 μM TTX, and the *I*_h_ residing in each cell was activated by stepping to −120 mV for a duration of 2 sec from a holding potential of −40 mV. As can be seen in Figure 3A,B, the subsequent addition of 10 μM oxaliplatin or 3 μM ivabradine, but still in continued exposure to 3 μM lutein, was able to attenuate or further decrease *I*_h_ amplitude, respectively. It is, therefore, clear that the *I*_h_ magnitude susceptible to being inhibited by lutein presented in GH_3_ cells would be effectively reversed by further application of oxaliplatin; however, it was further reduced by adding ivabradine.

### 2.4. Effect of Lutein on Voltage-Dependent Hysteresis (Hys_(V)_) of I_h_ Triggered by Inverted Isosceles-Triangular Ramp Voltage (V_ramp_)

**Figure 4:**
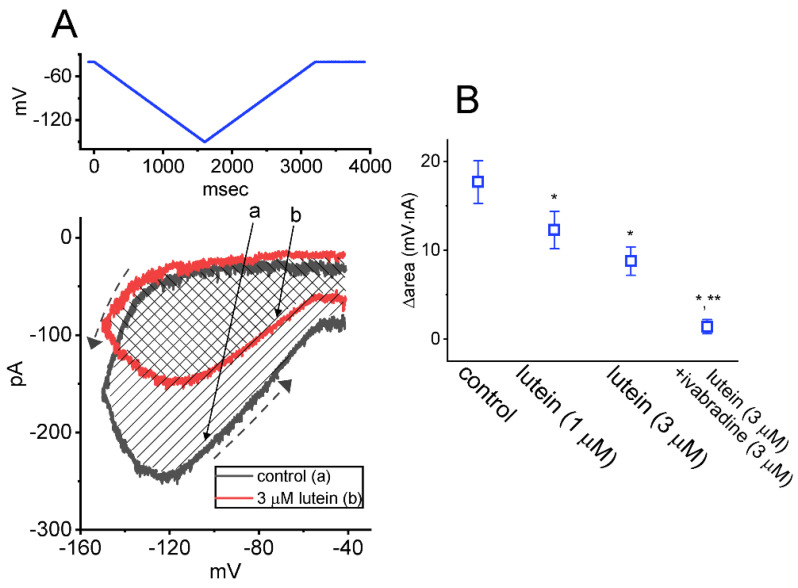
Modification by lutein on the strength in voltage-dependent hysteresis (Hys_(V)_) of *I*_h_ recorded from GH_3_ cells. (**A**) Representative Hys_(V)_ traces (i.e., the relationship of forward [descending] or reverse [ascending] current versus membrane potential) of *I*_h_ evoked by the inverted double (i.e., isosceles-triangular) V_ramp_ (indicated in the top part, blue color) in the absence (black color, a) or presence of 3 μM lutein (red color, b). The dashed black arrows along the current trace in the control period (i.e., lutein was not present) indicate an anti-clockwise direction of *I*_h_ trajectory in which time passes with the activation by inverted triangular V_ramp_, while the shaded region is the Hys_(V)_ area (∆area) with or without the lutein existence. (**B**) Summary graph revealing effects of lutein (1 or 3 μM) and lutein (3 μM) plus ivabradine (3 μM) on the ∆area of Hys_(V)_ (i.e., the shaded region under the curve activated during the downsloping and upsloping ends of triangular V_ramp_) of *I*_h_ (mean ± SEM; *n* = 7 for each point). Of note, there was an emergence of V_ramp_-induced Hys_(V)_ for *I*_h_ elicitation, and the presence of lutein was concentration-dependently able to produce a measurable reduction in the Hys_(V)_’s ∆area of the current. * Significantly different from control (*p* < 0.05) and ** significantly different from lutein-alone (3 μM) group (*p* < 0.05).

Previous observations disclosed the capability of *I*_h_ strength evoked by V_ramp_ to modify the varying patterns of bursting firing in different types of excitable cells, including central neurons [29,35,50,51,52]. Cyclic nucleotide-gated (CNG) channels (e.g., CNGA2 ion channels) were previously demonstrated to exhibit Hys_(V)_ behavior [53]. We hence continued to evaluate how lutein could have any propensity to adjust *I*_h_’s Hys_(V)_ responding to long-lasting triangular V_ramp_, the protocol of which was designed and then imposed on the tested cells through digital-to-analog conversion. In the current measurements, the tested cell was held at −40 mV, and we then applied a long-lasting inverted V_ramp_ to it. The V_ramp_ protocol comprises the downsloping (forward) ramp from −40 to −150 mV followed by the upsloping (backward) limb back to −40 mV with a total duration of 3.2 sec (i.e., a ramp speed of ±69 mV/sec) as indicated in the upper part of Figure 4A. As demonstrated in Figure 4, the voltage-dependent hysteresis (Hys_(V)_) of *I*_h_ (i.e., the relationship of forward or backward *I*_h_ versus membrane potential) was robustly observed upon activation by triangular double V_ramp_. That is, the *I*_h_ amplitude triggered by the downsloping limb of the inverted triangular V_ramp_ was overly lower than that by the upsloping end of V_ramp_. For example, in the control period (i.e., lutein was not present), the *I*_h_ amplitude at −120 mV taken during the downsloping or upsloping end of V_ramp_ was markedly different (*p* < 0.05) (i.e., 48 ± 8 pA [downsloping] versus 249 ± pA [upsloping]; *n* = 7, *p* < 0.05). Under cell exposure to lutein (3 μM), *I*_h_ evoked in the downsloping limb of the long-lasting inverted V_ramp_ was noticed to decline to a less extent than that measured from the upsloping end of the triangular V_ramp_. Moreover, the strength of lutein-induced current inhibition at the downsloping (forward) and upsloping (reverse) limbs of triangular V_ramp_ differ significantly. As demonstrated by the dashed arrows in Figure 4A, upon the difference (i.e., ∆area) in the shaded area under the curve in the downsloping and upsloping direction, we further quantified the degree of *I*_h_’s Hys_(V)_ residing in GH_3_ cells. The experimental observations disclosed that the amount of Hys_(V)_ responding to the long-lasting inverted V_ramp_ became conceivably decreased during cell exposure to lutein. Figure 4B summarized the data demonstrating the effects of lutein and lutein plus ivabradine on the ∆area (i.e., the shaded region in (A)) under such curve. For example, in addition to its depressive action on *I*_h_ magnitude, the addition of 3 μM lutein resulted in a reduction in the area responding to inverted triangular V_ramp_, as demonstrated by a reduction of ∆area from 17.7 ± 2.4 to 8.8 ± 1.6 mV·nA (*n* = 7, *p* < 0.05). After the lutein was removed, the Hys_(V)_’s area returned to 17.2 ± 2.3 mV·nA. As cells were continually exposed to 3 μM lutein, subsequent application of 3 μM ivabradine measurably decreased the ∆area of Hys_(V)_ further.

### 2.5. Mild Modification by Lutein of A-Type K^+^ Current (I_K(A)_) in GH_3_ Cells

**Figure 5:**
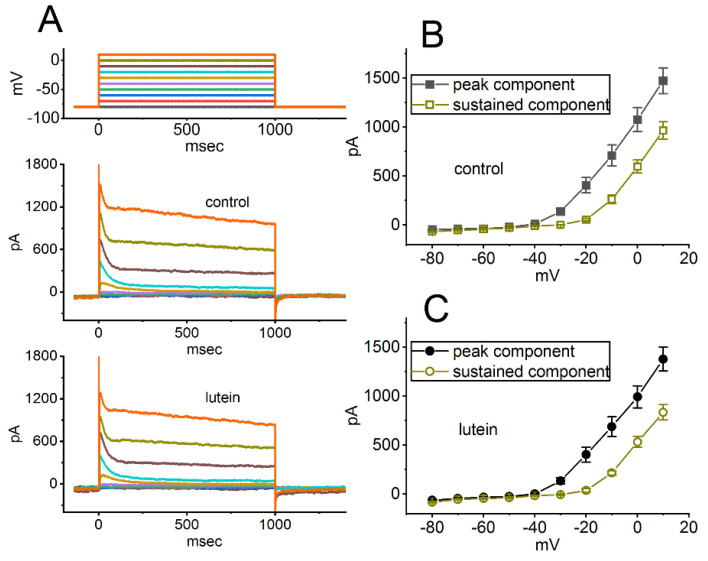
Effect of lutein on A-type K^+^ current (*I*_K(A)_) identified from GH_3_ cells. We conducted these experiments in cells bathed in Ca^2+^-free, Tyrode’s solution containing 1 μM TTX and 0.5 mM CdCl_2_, and the recording pipette was filled with K^+^-containing solution. (**A**) Superimposed current traces taken from the control period (i.e., lutein was not present; upper) and during exposure to 10 μM lutein (lower). The uppermost part indicates the voltage-clamp protocol imposed over the cell. The potential trace shown in different colors corresponds to the current one evoked by each membrane voltage. In (**B**,**C**), average *I*–*V* relationships of *I*_K(A)_ in the absence (**B**) and presence (**C**) of 10 μM lutein were demonstrated, respectively. The peak (filled black squares or circles) and sustained (open brown squares or circles) components of *I*_K(A)_ in (**B**,**C**) were respectively measured at the beginning and end of each voltage step. Each point represents the mean ± SEM (*n* = 7 for each point).

We also continued to study if the presence of lutein could influence macroscopic K^+^ currents (e.g., *I*_K(A)_) identified in these cells. These experiments were conducted in cells which were bathed in Ca^2+^-free, Tyrode’s solution containing 1 μM TTX, and we filled up the recording pipette by adding K^+^-enriched (145 mM) solution. As depicted in Figure 5A, with cell exposure to lutein at a concentration of 10 μM, the amplitudes of *I*_K(A)_ were robustly reduced, which were activated upon different levels of voltage steps with a rapidly activating and slowing inactivating time course of the current [54,55]. The average *I–V* relationship of peak and sustained *I*_K(A)_ in the control period (i.e., lutein was not present) or during cell exposure to 10 μM lutein was collated and is hence illustrated in Figure 5B or Figure 5C, respectively. For example, cell exposure to 10 μM lutein markedly decreased the peak *I*_K(A)_ at 0 mV from 1076 ± 122 to 992 ± 112 pA (*n* = 7, *p* < 0.05). Therefore, the presence of lutein (10 μM) brought about a mild suppression in the peak and sustained amplitudes of *I*_K(A)_ with no marked change in the inactivation time course of the current.

### 2.6. Effect of Lutein on erg-Mediated K^+^ Current (I_K(erg)_) in GH_3_ Cells

**Figure 6:**
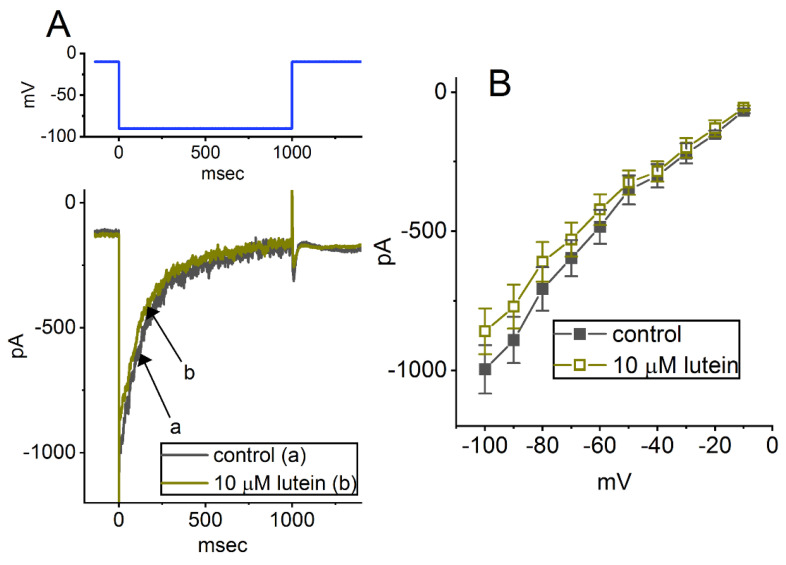
Effect of lutein on *erg*-mediated K^+^ current (*I*_K(erg)_) recorded from GH_3_ cells. In these experiments of amplifying *I*_K(erg)_, we placed cells in high-K^+^ (145 mM), Ca^2+^-free solution, and the recording pipette was filled with K^+^-containing solution. (**A**) Representative current traces activated by the hyperpolarizing step from −10 to −90 mV (indicated in the upper part, blue color). Current trace labeled a is the control (i.e., lutein was not present, black color), and that labeled b was recorded during the exposure of 10 μM lutein (brown color). (**B**) Average *I–V* relationship of *I*_K(erg)_ taken in the absence (filled black squares) and presence (open brown squares) of 10 μM lutein (mean ± SEM; *n* = 7 for each point). Current amplitude (i.e., deactivating *I*_K(erg)_) was acquired at the beginning of each voltage step from a holding potential of −10 mV.

We also explored whether cell exposure to lutein could modify another type of K^+^ currents (e.g., *I*_K(erg)_). To amplify *I*_K(erg)_, we placed cells in a high-K^+^, Ca^2+^-free solution, and the recording pipette was filled with K^+^-containing solution. As shown in Figure 6A, when we held the tested cells at −10 mV, and a 1 sec hyperpolarizing step to −90 mV was imposed to activate the deactivating *I*_K(erg)_ with a slowly decaying time course [56,57,58]. One minute after cells were exposed to 10 μM lutein, upon membrane hyperpolarization from −10 to −90 mV, the peak amplitude of *I*_K(erg)_ decreased from 891 ± 83 to 771 ± 79 pA (*n* = 7, *p* < 0.05). After washout of the compound, *I*_K(erg)_ amplitude returned to 885 ± 22 pA (*n* = 7). The average *I–V* relationship of peak *I*_K(erg)_ with or without cell exposure to 10 μM lutein was constructed and is hence illustrated in Figure 6B. Therefore, similar to the results described above in *I*_K(A)_, the data showed that lutein led to a mild inhibitory effect on *I*_K(erg)_ activated in response to long-lasting membrane hyperpolarization.

### 2.7. Effect of Lutein on Sag Potential Measured from GH_3_ Cells

**Figure 7:**
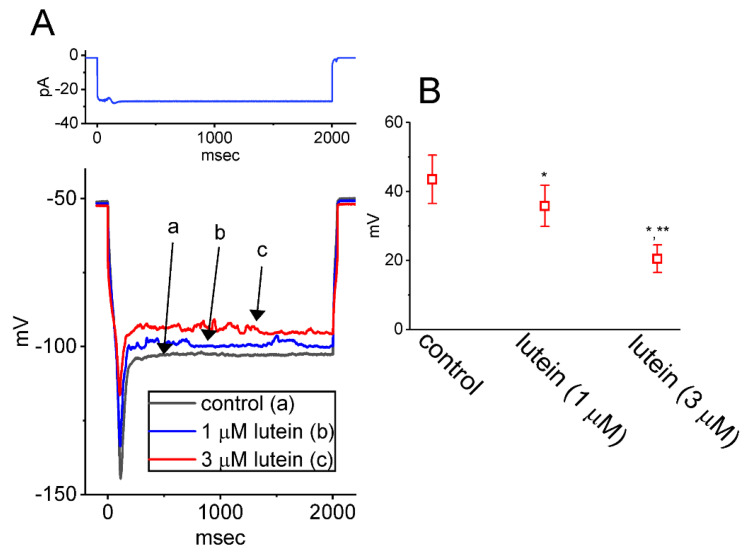
Effect of lutein on sag potential recorded from GH_3_ cells. In this set of current-clamp potential recordings, we bathed cells in normal Tyrode’s solution, which contained 1.8 mM CaCl_2_ and 1 μM TTX, and a long-last hyperpolarizing current stimulus with a duration of 2 sec was then applied to the tested cell. (**A**) Representative potential traces acquired in the control period (a, black color) and during cell exposure to 1 μM lutein (b, blue color) or 3μM lutein (c, red color). The top part indicates the long-step current injection imposed over the tested cell. (**B**) Summary scatter graph demonstrating the capability of lutein to alter the amplitude of sag potential (mean ± SEM; *n* = 7 for each point). Under the current-clamp configuration, the magnitude of sag potential (i.e., the difference between the start and end of hyperpolarizing current stimulus) was acquired with or without cell exposure to lutein (1 or 3 μM). * Significantly different from control (*p* < 0.05) and ** significantly different from the lutein-alone (1 μM) group (*p* < 0.05).

We further switched to current-clamp potential recordings in an attempt to examine if lutein could result in any changes on sag potential in these cells. Sag potential evoked in response to hyperpolarizing current stimulus has been demonstrated previously to be linked to the occurrence of *I*_h_ in different types of excitable cells [59,60,61]. In this set of experiments, GH_3_ cells were placed in normal Tyrode’s solution containing 1.8 mM CaCl_2_, and the pipette was filled with K^+^-enrich solution. Current-clamp configuration with a holding current of 0 pA was then established. As shown in Figure 7, under our experimental conditions, when the whole-cell potential recordings were achieved, a long-step 2-sec hyperpolarizing current injection with the amplitude of around 25 pA was strikingly noticed to induce the occurrence of sag potential (i.e., an abrupt drop-down to a lower level in the membrane potential upon hyperpolarizing current injection) [62]. Cell exposure to ivabradine (3 μM) was effective at suppressing the amplitude of sag potential. Furthermore, the application of 1 or 3 μM lutein resulted in a conceivable depression of sag potential evoked in response to hyperpolarizing current stimulus. For example, lutein at a concentration of 3 μM measurably decreased the amplitude of sag potential from 43.5 ± 7.0 to 20.5 ± 34.9 mV (*n* = 7, *p* < 0.05). It is conceivable, therefore, that the sag potential presented in GH_3_ cells is associated with the magnitude of *I*_h_ and that the depression of such potential produced by the presence of lutein could be largely ascribed from its inhibitory effectiveness in the *I*_h_ amplitude of these cells.

## 3. Discussion

The principal findings in this work are that GH_3_-cell exposure to lutein is capable of suppressing *I*_h_ in a concentration-, state-, voltage-, and Hys_(V)_-dependent manner. A hyperpolarizing shift of the steady-state activation curve of *I*_h_ was observed in the presence of this compound. The strength of V_ramp_-evoked Hys_(V)_ was also decreased by adding it. Under current-clamp configuration, the amplitude of sag potential observed in GH_3_ cells was also decreased during exposure to lutein. HCN2, HCN3, or mixed HCN2+HCN3 channels that can be encoded for generation of macroscopic *I*_h_ were reported to be distributed in pituitary GH_3_ cells [27,28,39]. Mild inhibition of *I*_K(erg)_ or *I*_K(A)_ by adding lutein was observed in this study. Under the current-clamp configuration, the exposure to lutein can result in a decrease in the magnitude of sag potential. Together, findings from the present study can thus be interpreted to mean that, besides its antioxidative or anti-inflammatory properties, the existence of lutein can inhibit the amplitude as well as alter gating and Hys_(V)_ behavior of *I*_h_, and that lutein’s actions presented herein would engage in the modifications on spontaneous actions potentials present in electrically excitable cells (e.g., GH_3_ cells), presuming that similar in vivo observations occur.

In this study, a left shift in the steady-state activation curve of *I*_h_ with no concurrent modifications in the number of apparent gating charge in the curve was demonstrated with the existence of lutein. The τ_act_ value estimated from the slowly activating *I*_h_ during cell exposure to lutein also turned out to be larger because of a concurrent slowing in the activation time course of the current. Cell exposure to lutein was thus capable of altering hyperpolarization-activated *I*_h_ in a time-, concentration- and voltage-dependent manner. Whatever the ionic mechanism of the active lutein, these results can be interpreted to mean that the responsiveness in different cell types to this compound would heavily rely either on the lutein concentration achieved, pre-existing levels of resting membrane potential, different patterns of action potential firing, or a combination of the three.

The Hys_(V)_ properties of *I*_h_ activated in response to triangular V_ramp_ have been viewed to play a role in perturbing the electrical behaviors of various excitable cells [29,38,50,51,53,63]. Voltage-sensing domain relaxation has also been noticed to have a unique impact on such Hys_(V)_ behavior observed in the proteins (i.e., HCNx channels) [29,50,64,65]. In other words, the observed “inertia” in the responsiveness of HCNx channels can be driven by changes in their electrical sensitivity, which was allowed to resemble that of ferromagnetic materials displaying Hys_(V)_ behaviors [52,66]. Of considerable interest, in accordance with previous observations [29,49,63], the *I*_h_ intrinsically residing in GH_3_ cells was noticed to undergo a non-equilibrium property of instantaneous *I*_h_—that is, an anti-clockwise Hys_(V)_ loop responding to the isosceles-triangular V_ramp_. Such change has been viewed to be dynamically linked to a mode shift in situations where the voltage sensitivity of gating charge movements (i.e., voltage-sensing domain relaxation) relies on the previous state (or conformation) of the channel [50,52,63,64]. Notably, GH_3_-cell exposure to lutein presented herein resulted in a significant reduction in Hys_(V)_ strength (i.e., shaded region in Figure 4) of *I*_h_ evoked by long-lasting triangular V_ramp_. However, whether the lutein molecule can interact with the voltage-sensing domains of HCNx channels remains to be further delineated.

In previous studies, different HCN isoforms have been demonstrated to combine to constitute macroscopic *I*_h_ existing in different types of excitable cells [26,28,67]. Because HCN2, HCN3, or mixed HCN2+HCN3 channels were reported to be functionally expressed in GH_3_ cells [28,39], it appears unlikely that lutein-induced block of *I*_h_ inherently in native cells is isoform-specific. Of additional note, it has been reported that the blocker of HCN channels could be linked to changes in the phosphene perception of the retina [68], which might potentially account for lutein-mediated amelioration in the retina (e.g., macular degeneration) [4,7,8,10,37].

Earlier investigations have shown that the cyclic nucleotide-gated (CNG) channels, the behavior of which was noticed to show Hys_(V)_ behavior in their gating mechanism, are regarded to mediate receptor potentials involved in olfaction and vision [53]. The HCN channels, another family of ionic channels gated by cyclic nucleotides, have been previously demonstrated to be linked to phototransduction in photosensitive retinal ganglion cells [47]. Its activity was found to alter the electroretinographic ON and OFF responses or to delay photoreceptor degeneration [19,68,69,70]. To what extent do lutein-mediated changes in magnitude, gating kinetics and Hys_(V)_ behavior of *I*_h_ presented herein engage in lutein-mediated action on age-related diseases (e.g., macular degeneration or retinitis pigmentosa) [8,19,71,72,73,74] still needs to be further delineated.

In this study, the lutein concentration required for the half-maximal inhibition (i.e., IC_50_) of *I*_h_ detected in GH_3_ cells was calculated to be 4.1 μM. From the previous pharmacokinetic studies, it has been previously reported that plasma lutein concentration could reach around 2.5 μM after daily doses of lutein (20.5 mg) for 42 days, and that it could increase 3.5- and 10-fold on average, respectively, after the long-term intake of 4.1 and 20.5 mg lutein [75]. In comparison, it is, therefore, reasonable to assume that the observed effects by lutein on the amplitude, gating and Hys_(V)_ of *I*_h_ (or HCNx-encoded currents) may achieve the concentration of therapeutic or pharmacological requirements [75,76].

**Figure 8:**
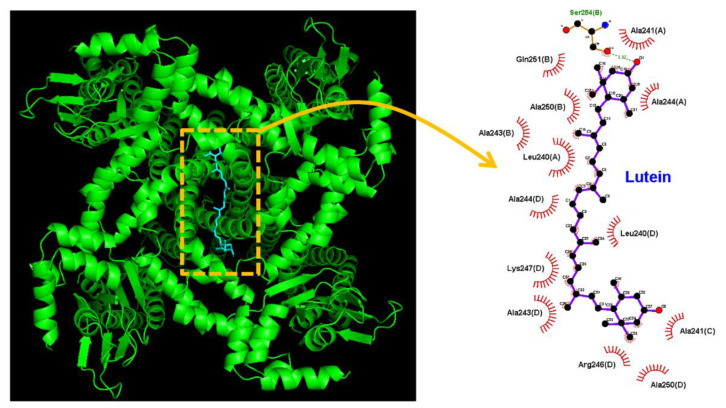
Docking results of HCN channel and the lutein molecule. The protein structure of HCN channel was acquired from RCSB PDB (PDB ID: 5V4S), while the lutein structure was from PubChem (Compound CID: 5281243). The structure of the HCN channel was docked with the lutein molecule through PyRx software (https://pyrx.sourceforge.io/) (accessed on 1 June 2022). The diagram of the interaction between the HCN channel and the lutein molecule was generated by LigPlot^+^ (https://www.ebi.ac.uk/thornton-srv/software/LIGPLOT/) (accessed on 1 June 2022). Of note, the red arcs with spokes radiating toward the ligand (i.e., lutein) indicate hydrophobic contacts, whereas the green dot line depicts hydrogen bond.

In this study, we additionally investigated how the protein of HCN channel could be docked with the lutein molecule through PyRx software. The protein structure of HCN channel was acquired from PDB (PDB ID: 5V4S) [77]. The predicted binding sites of the lutein with which the amino-acid residues can interact are presented in Figure 8. It should be noted that the lutein molecule may form hydrophobic contacts with residue Leu240(A), Leu240(D), Ala241(A), Ala241(C), Ala243(B), Ala243(D), Ala244(A), Ala244(D), Arg246(D), Lys247(D), Ala250(B), Ala250(D), and Gln251(B), while it has a hydrogen bond with residue Ser254(B) with a distance of approximately 3.07 Å. These results prompted us to reflect that the lutein molecule can bind to the transmembrane segment (position: 242–269) of HCN channels (PDB: 5V4S), the site of which is different to a certain extent from those reported recently [43]. It is thus likely that the interaction of the lutein molecules with HCN channels could be located at the cytosolic side of the membrane, although the detailed ionic mechanisms of lutein’s actions are currently unknown.

## 4. Materials and Methods

### 4.1. Chemicals, Drugs and Solutions Used in the Present Work

Lutein (xanthophyll, Yè huáng sù, β,ε-carotene-3,3′-diol, 3,3′-di-hydroxy-β,ε-carotene, (1R)-4-[(1E,3E,5E,7E,9E,11E,13E,15E,17E)-18-[(1R,4R)-4-hydroxy-2,6,6-trimethylcyclohex-2-en-1-yl]-3,7,12,16-tetramethyloctadeca-1,3,5,7,9,11,13,15,17-nonaenyl]-3,5,5-trimethylcyclohex-3-en-1-ol, CID 5281243, C40H56O2, https://pubchem.ncbi.nlm.nih.gov/compound/5281243) (accessed on 1 June 2022) was acquired from MedChemExpress (Genechain, Kaohsiung, Taiwan), while ivabradine, oxaliplatin and tetrodotoxin (TTX) were from Sigma-Aldrich (St. Louis, MO). Lutein was dissolved in dimethyl sulfoxide at a concentration of 10 mM and made immediately before experiments. To protect lutein from being degraded by light [7], stock solution containing this compound was wrapped in aluminum foil, and it was kept under −20 °C for long-term storage. For cell preparations, we acquired fetal bovine and calf serum, L-glutamine, trypsin/EDTA, and cell culture media from HycloneTM (Thermo Fisher; Level Biotech, Tainan, Taiwan). Unless stated otherwise, other chemicals, reagents, or solvents were of analytical reagent grade and supplied by Sigma-Aldrich.

The HEPES-buffered normal Tyrode’s solution contained (in mM): NaCl 136.5, KCl 5.4, MgCl_2_ 0.53, CaCl_2_ 1.8, glucose 5.5, HEPES 5.5, and the pH was adjusted to 7.4 by adding NaOH. For measurements of *I*_h_ or *I*_K(A)_ across the cell membrane, GH_3_ cells were placed in Ca^2+^-free, Tyrode’s solution in order to avoid the contamination of voltage-gated Ca^2+^ currents and Ca^2+^-activated K^+^ currents. To record *I*_K(erg)_, we bathed cells in high-K^+^, Ca^2+^-free solution containing: KCl 145, MgCl_2_ 0.53 and HEPES-KOH 5 (in mM and pH 7.4). In whole-cell current or potential recordings, we filled up the recording pipette with an internal solution, which contained: K-aspartate 140, KH_2_PO_4_ 1, Na_2_ATP 3, Na_2_GTP 0.1, EGTA 0.1, and HEPES 4 (in mM), and the pH was adjusted to 7.4 with KOH. The chemicals or reagents used to make these solutions were acquired from Sigma-Aldrich. The twice-distilled water was deionized through a Millipore-Q system and used in all experiments.

### 4.2. Cell Preparations

Pituitary GH_3_ somatolactotrophs, originally supplied by the Bioresources Collection and Research Center ([BCRC-60015, http://catalog.bcrc.firdi.org.tw/BcrcContent?bid=60015] (accessed on 1 June 2022), Hsinchu, Taiwan), were maintained in Ham’s F-12 medium, with which 15% heat-inactivated horse serum (*v*/*v*), 2.5% fetal calf serum (*v*/*v*), and 2 mM L-glutamine were supplemented. They were grown in monolayer culture in 50-mL plastic culture flasks in a humidified environment of CO_2_/air (1:19). Subcultures were made by trypsinization (0.025% trypsin solution [HyClone^TM^] containing 0.01% sodium *N*,*N*-diethyldithiocarbamate and EDTA). We carried out electrophysiological measurements 5 or 6 days after GH_3_ cells underwent subculture (60–70% confluence).

### 4.3. Electrophysiological Measurements

Shortly before the recordings, we carefully dispersed the GH_3_ with 1% trypsin-EDTA solution, and thereafter placed a few aliquots of cell suspension containing clumps of cells into a home-made chamber tightly mounted on the working stage of a DM-II inverted microscope (Leica; Major Instruments, Tainan, Taiwan). Cells were immersed at room temperature (20–25 °C) in HEPES-buffered normal Tyrode’s solution, ionic composition of which was elaborated above. The electrode that we used to record were prepared from Kimax^®^-51 capillaries with 1.5–1.8 mm outer diameter (#34500; Kimble; Dogger, New Taipei City, Taiwan) by using a vertical puller (PP-83; Narishige, Major Instruments). Being filled with different internal solutions stated above, the recording electrodes had tip resistances of 3–5 MΩ. We measured ionic currents or membrane potential in the whole-cell current or potential recording of a modified patch-clamp technique, respectively, by using either a MultiClamp 700B (Molecular Devices, Sunnyvale, CA) or an RK-400 amplifier (Bio-Logic, Claix, France), as described elsewhere [24,49,55]. GΩ-seals were established in an all-or-nothing fashion and hence resulted in a dramatic improvement in the signal-to-noise ratio. The liquid junction potential, which occurred when the ionic compositions in the pipette solution and those of the external solution were different, was zeroed shortly before GΩ-seal formation was made, and the whole-cell data were then corrected. During measurements, we exchanged the solution through a homemade gravity-driven type of bath perfusion. In order to measure the *I*_h_, we voltage-clamped the examined cell at a holding potential of −40 mV before a long-lasting hyperpolarizing step was delivered.

The signals, comprising both potential and current traces, were monitored at a given interval and digitally stored online at 10 kHz in an ASUS ExpertBook laptop computer (P2451F; Yuan-Dai, Tainan, Taiwan). For efficient analog-to-digital (A/D) and digital-to-analog (D/A) conversion to proceed during the measurements, a low-noise Digidata^®^ 1440A equipped with an ASUS computer was controlled by pClamp^TM^ 10.6 software run under Microsoft Windows 7 (Redmond, WA, USA). We low-pass filtered current signals at 2 kHz with a FL-4 four-pole Bessel filter (Dagan, Minneapolis, MN, USA). A variety of pClamp-generated voltage-clamp protocols with various rectangular or ramp waveforms were designed, and they were then applied to the tested cells through D/A conversion in attempts to assess the current–voltage (*I*–*V*) relation or steady-state activation curve of the currents.

### 4.4. Data Analyses

To assess concentration-dependent inhibition of lutein on *I*_h_ amplitude, we put GH_3_ cells in Ca^2+^-free Tyrode’s solution. During the measurements, we voltage-clamped each cell at a holding potential of −40 mV and a long-lasting hyperpolarizing pulse to −120 mV for a duration of 2 sec was delivered to evoke *I*_h_. The *I*_h_ amplitudes were thereafter measured at the end pulse of 2-sec hyperpolarizing command voltage during cell exposure to different lutein concentrations (0.1–100 μM). The concentration-dependent inhibition by lutein of *I*_h_ in GH_3_ cells was determined by fitting a modified Hill function. That is,

Percentage decrease %=Emax×luteinnHIC50nH+luteinnH

where [*lutein*] = the lutein concentration applied; *n_H_* = the Hill coefficient; *IC*_50_ = the concentration required for a 50% inhibition of *I*_h_ amplitude activated by long-lasting hyperpolarizing step; *E_max_* = lutein-induced maximal inhibition of *I*_h_.

To assess lutein-induced modifications on the quasi-steady-state activation of *I*_h_, the relationship between the normalized amplitude of *I*_h_ and the conditioning potential acquired with or without cell exposure to 3 μM lutein was constructed, and the data were thereafter fitted by chi-squared goodness of fit with a Boltzmann function of the following form:

I=Imax1+exp−V−V12RT/qF

where *I*_max_ = the maximal *I*_h_ magnitude acquired with or without cell exposure to the lutein (3 μM) at the conditioning potential of −30 mV; *V*_1/2_ = the voltage at which half-maximal activation (i.e., midpoint potential) occurs; *q* = the apparent gating charge; *F* = the Faraday constant, *R* the idea gas constant; *T* = the absolute temperature.

### 4.5. Curve-Fitting Approximations and Statistical Analyses

Curve-fitting (linear or non-linear [e.g., Hill or Boltzmann equation] to experimental data sets was dealt with the goodness of fit by using different analytical procedures, which include the Microsoft “Solver” built into Excel^®^ 2019 (Microsoft) and OriginPro^®^ 2021 program (Microcal; Scientific Formosa, Kaohsiung, Taiwan). The electrophysiological data (whole-cell current or potential data) are presented as the mean ± standard error of the mean (SEM), with the size of experimental observations (*n*) indicative of cell numbers from which the data were obtained. The data distribution was found to satisfy the tests for normality. We performed Student’s *t*-tests (for paired or unpaired samples) between the two different groups, while for more than two different groups, analysis of variance (for one-way ANOVA or two-way ANOVA) with or without repeated measured followed by a post-hoc Fisher’s least-significance difference test was utilized to determine the significance. A *p*-value of less than 0.05 was considered to indicate statistical difference (marked with * or ** in the figures).

## 5. Conclusions

These results reveal that lutein profoundly leads to a reduction in hyperpolarization-evoked *I*_h_ (or HCNx-encoded currents) in a concentration-, time-, voltage-, and in a Hys_(V)_-dependent manner. Our findings on GH_3_ cells thus shed light on the evidence revealing the effectiveness of lutein in modifying specific ionic currents (e.g., *I*_h_), and these data may be linked to its additional effects, which are potentially useful pharmacological applications in different excitable cells occurring in vivo.

## Data Availability

The original data is available upon reasonable request to the corresponding author.

## Abbreviations

ANOVA	analysis of variance
CNG channel	cyclic nucleotide-gated channel
HCN channel	hyperpolarization-activated cyclic nucleotide-gated channel
IC_50_	concentration required for 50% inhibition (half-maximal inhibitory concentration)
*I*_h_	hyperpolarization-activated cation current
*I*_K(A)_	A-type K^+^ current
*I*_K(erg)_	*erg*-mediated K^+^ current
OXAL	oxaliplatin
SEM	standard error of mean
τ_act_	activation time constant
TTX	tetrodotoxin
Hys_(V)_	voltage-dependent hysteresis
V_ramp_	ramp voltage

## References

[1] Calvo M.M. (2005). Lutein: A valuable ingredient of fruit and vegetables. Crit. Rev. Food Sci. Nutr..

[2] Izumi-Nagai K., Nagai N., Ohgami K., Satofuka S., Ozawa Y., Tsubota K., Umezawa K., Ohno S., Oike Y., Ishida S. (2007). Macular pigment lutein is antiinflammatory in preventing choroidal neovascularization. Arterioscler. Thromb. Vasc. Biol..

[3] Liu R.H. (2013). Health-promoting components of fruits and vegetables in the diet. Adv. Nutr..

[4] Li L.H., Lee J.C., Leung H.H., Lam W.C., Fu Z., Lo A.C.Y. (2020). Lutein Supplementation for Eye Diseases. Nutrients.

[5] Mitra S., Rauf A., Tareq A.M., Jahan S., Emran T.B., Shahriar T.G., Dhama K., Alhumaydhi F.A., Aljohani A.S.M., Rebezov M. (2021). Potential health benefits of carotenoid lutein: An updated review. Food Chem. Toxicol..

[6] Johnson E.J. (2014). Role of lutein and zeaxanthin in visual and cognitive function throughout the lifespan. Nutr. Rev..

[7] Kijlstra A., Tian Y., Kelly E.R., Berendschot T.T. (2012). Lutein: More than just a filter for blue light. Prog. Retin. Eye Res..

[8] Bernstein P.S., Li B., Vachali P.P., Gorusupudi A., Shyam R., Henriksen B.S., Nolan J.M. (2016). Lutein, zeaxanthin, and meso-zeaxanthin: The basic and clinical science underlying carotenoid-based nutritional interventions against ocular disease. Prog. Retin. Eye Res..

[9] Manayi A., Abdollahi M., Raman T., Nabavi S.F., Habtemariam S., Daglia M., Nabavi S.M. (2016). Lutein and cataract: From bench to bedside. Crit. Rev. Biotechnol..

[10] Eisenhauer B., Natoli S., Liew G., Flood V.M. (2017). Lutein and Zeaxanthin-Food Sources, Bioavailability and Dietary Variety in Age-Related Macular Degeneration Protection. Nutrients.

[11] Jia Y.P., Sun L., Yu H.S., Liang L.P., Li W., Ding H., Song X.B., Zhang L.J. (2017). The Pharmacological Effects of Lutein and Zeaxanthin on Visual Disorders and Cognition Diseases. Molecules.

[12] Long A.C., Kuchan M., Mackey A.D. (2019). Lutein as an Ingredient in Pediatric Nutritionals. J. AOAC Int..

[13] Hayashi R., Hayashi S., Machida S. (2022). Changes in Aqueous Humor Lutein Levels of Patients with Cataracts after a 6-Week Course of Lutein-Containing Antioxidant Supplementation. Curr. Eye Res..

[14] Nakamura M., Sugiura M. (2022). Serum Lutein and Zeaxanthin Are Inversely Associated with High-Sensitivity C-Reactive Protein in Non-Smokers: The Mikkabi Study. Antioxidants.

[15] Al-Rafiah A.R., Mehdar K.M. (2021). Histopathological and Biochemical Assessment of Neuroprotective Effects of Sodium Valproate and Lutein on the Pilocarpine Albino Rat Model of Epilepsy. Behav. Neurol..

[16] Lopresti A.L., Smith S.J., Drummond P.D. (2022). The Effects of Lutein and Zeaxanthin Supplementation on Cognitive Function in Adults With Self-Reported Mild Cognitive Complaints: A Randomized, Double-Blind, Placebo-Controlled Study. Front. Nutr..

[17] Yuceli S., Yazici G.N., Mammadov R., Suleyman H., Ozdogan (2021). S. The Effect of Lutein on Ischemia-reperfusion-induced Vasculitic Neuropathic Pain and Neuropathy in Rats. Vivo.

[18] Hanaguri J., Yokota H., Kushiyama A., Kushiyama S., Watanabe M., Yamagami S., Nagaoka T. (2022). Beneficial Effect of Long-Term Administration of Supplement With Trapa Bispinosa Roxb. and Lutein on Retinal Neurovascular Coupling in Type 2 Diabetic Mice. Front. Physiol..

[19] Zhang H.J., Liu X.B., Chen X.M., Kong Q.H., Liu Y.S., So K.F., Chen J.S., Xu Y., Mi X.S., Tang S.B. (2022). Lutein delays photoreceptor degeneration in a mouse model of retinitis pigmentosa. Neural. Regen. Res..

[20] Al-Ishaq R.K., Overy A.J., Büsselberg D. (2020). Phytochemicals and Gastrointestinal Cancer: Cellular Mechanisms and Effects to Change Cancer Progression. Biomolecules.

[21] Han L., Song X. (2022). Lutein induces an inhibitory effect on the malignant progression of pancreatic adenocarcinoma by targeting BAG3/cholesterol homeostasis. J. Biochem. Mol. Toxicol..

[22] Georgiou C.D., Margaritis L.H. (2021). Oxidative Stress and NADPH Oxidase: Connecting Electromagnetic Fields, Cation Channels and Biological Effects. Int. J. Mol. Sci..

[23] Irisawa H., Brown H.F., Giles W. (1993). Cardiac pacemaking in the sinoatrial node. Physiol. Rev..

[24] Simasko S.M., Sankaranarayanan S. (1997). Characterization of a hyperpolarization-activated cation current in rat pituitary cells. Am. J. Physiol..

[25] Ono K., Shibata S., Iijima T. (2003). Pacemaker mechanism of porcine sino-atrial node cells. J. Smooth Muscle Res..

[26] DiFrancesco D. (2006). Serious workings of the funny current. Prog. Biophys. Mol. Biol..

[27] Kretschmannova K., Gonzalez-Iglesias A.E., Tomić M., Stojilkovic S.S. (2006). Dependence of hyperpolarisation-activated cyclic nucleotide-gated channel activity on basal cyclic adenosine monophosphate production in spontaneously firing GH3 cells. J. Neuroendocrinol..

[28] Kretschmannova K., Kucka M., Gonzalez-Iglesias A.E., Stojilkovic S.S. (2012). The expression and role of hyperpolarization-activated and cyclic nucleotide-gated channels in endocrine anterior pituitary cells. Mol. Endocrinol..

[29] Xiao Y.F., Chandler N., Dobrzynski H., Richardson E.S., Tenbroek E.M., Wilhelm J.J., Sharma V., Varghese A., Boyett M.R., Iaizzo P.A. (2010). Hysteresis in human HCN4 channels: A crucial feature potentially affecting sinoatrial node pacemaking. Sheng Li Xue Bao.

[30] Calejo A.I., Jorgačevski J., Rituper B., Guček A., Pereira P.M., Santos M.A., Potokar M., Vardjan N., Kreft M., Gonçalves P.P. (2014). Hyperpolarization-activated cyclic nucleotide-gated channels and cAMP-dependent modulation of exocytosis in cultured rat lactotrophs. J. Neurosci..

[31] Lee C.H., MacKinnon R. (2017). Structures of the Human HCN1 Hyperpolarization-Activated Channel. Cell.

[32] Zobeiri M., Chaudhary R., Blaich A., Rottmann M., Herrmann S., Meuth P., Bista P., Kanyshkova T., Lüttjohann A., Narayanan V. (2019). The Hyperpolarization-Activated HCN4 Channel is Important for Proper Maintenance of Oscillatory Activity in the Thalamocortical System. Cereb. Cortex.

[33] Li D., Liu H., Liu X., Wang H., Li T., Wang X., Jia S., Wang P., Wang Y.F. (2020). Involvement of Hyperpolarization-Activated Cyclic Nucleotide-Gated Channel 3 in Oxytocin Neuronal Activity in Lactating Rats with Pup Deprivation. ASN Neuro.

[34] Santoro B., Shah M.M. (2020). Hyperpolarization-Activated Cyclic Nucleotide-Gated Channels as Drug Targets for Neurological Disorders. Annu. Rev. Pharmacol. Toxicol..

[35] Wei F., Wang Q., Han J., Goswamee P., Gupta A., McQuiston A.R., Liu Q., Zhou L. (2020). Photodynamic Modification of Native HCN Channels Expressed in Thalamocortical Neurons. ACS Chem. Neurosci..

[36] Wu S.-N., Fang Y.H., Liu P.Y., Liu Y.W. (2020). Characterization of hyperpolarization-induced cation current in differentiated human embryonic stem cell-derived cardiomyocytes. J. Am. Coll. Cardiol..

[37] Barravecchia I., Demontis G.C. (2021). HCN1 channels: A versatile tool for signal processing by primary sensory neurons. Prog. Biophys. Mol. Biol..

[38] Peters C.H., Liu P.W., Morotti S., Gantz S.C., Grandi E., Bean B.P., Proenza C. (2021). Bidirectional flow of the funny current (I(f)) during the pacemaking cycle in murine sinoatrial node myocytes. Proc. Natl. Acad. Sci. USA.

[39] Liu Y.C., Wang Y.J., Wu P.Y., Wu S.N. (2009). Tramadol-induced block of hyperpolarization-activated cation current in rat pituitary lactotrophs. Naunyn Schmiedebergs Arch. Pharmacol..

[40] Hsiao H.T., Liu Y.C., Liu P.Y., Wu S.N. (2019). Concerted suppression of I(h) and activation of I(K(M)) by ivabradine, an HCN-channel inhibitor, in pituitary cells and hippocampal neurons. Brain Res. Bull..

[41] Yang L., Liu X., Yao K., Sun Y., Jiang F., Yan H., Mao P., Fan S., Wei X., Liu Y. (2019). HCN channel antagonist ZD7288 ameliorates neuropathic pain and associated depression. Brain Res..

[42] Lu T.L., Lu T.J., Wu S.N. (2020). Inhibitory Effective Perturbations of Cilobradine (DK-AH269), A Blocker of HCN Channels, on the Amplitude and Gating of Both Hyperpolarization-Activated Cation and Delayed-Rectifier Potassium Currents. Int. J. Mol. Sci..

[43] Nakashima K., Nakao K., Matsui H. (2021). Discovery of Novel HCN4 Blockers with Unique Blocking Kinetics and Binding Properties. SLAS Discov..

[44] Benarroch E.E. (2013). HCN channels: Function and clinical implications. Neurology.

[45] Chang X., Wang J., Jiang H., Shi L., Xie J. (2019). Hyperpolarization-Activated Cyclic Nucleotide-Gated Channels: An Emerging Role in Neurodegenerative Diseases. Front. Mol. Neurosci..

[46] Combe C.L., Gasparini S. (2021). I(h) from synapses to networks: HCN channel functions and modulation in neurons. Prog. Biophys. Mol. Biol..

[47] Jiang Z., Yue W.W.S., Chen L., Sheng Y., Yau K.W. (2018). Cyclic-Nucleotide- and HCN-Channel-Mediated Phototransduction in Intrinsically Photosensitive Retinal Ganglion Cells. Cell.

[48] Resta F., Micheli L., Laurino A., Spinelli V., Mello T., Sartiani L., Di Cesare Mannelli L., Cerbai E., Ghelardini C., Romanelli M.N. (2018). Selective HCN1 block as a strategy to control oxaliplatin-induced neuropathy. Neuropharmacology.

[49] Chang W.T., Gao Z.H., Li S.W., Liu P.Y., Lo Y.C., Wu S.N. (2020). Characterization in Dual Activation by Oxaliplatin, a Platinum-Based Chemotherapeutic Agent of Hyperpolarization-Activated Cation and Electroporation-Induced Currents. Int. J. Mol. Sci..

[50] Männikkö R., Pandey S., Larsson H.P., Elinder F. (2005). Hysteresis in the voltage dependence of HCN channels: Conversion between two modes affects pacemaker properties. J. Gen. Physiol..

[51] Barthel L., Reetz O., Strauss U. (2016). Use Dependent Attenuation of Rat HCN1-Mediated Ih in Intact HEK293 Cells. Cell. Physiol. Biochem..

[52] Villalba-Galea C.A., Chiem A.T. (2020). Hysteretic Behavior in Voltage-Gated Channels. Front. Pharmacol..

[53] Nache V., Eick T., Schulz E., Schmauder R., Benndorf K. (2013). Hysteresis of ligand binding in CNGA2 ion channels. Nat. Commun..

[54] Simasko S.M. (1991). Evidence for a delayed rectifier-like potassium current in the clonal rat pituitary cell line GH3. Am. J. Physiol..

[55] Cho H.Y., Chuang T.H., Wu S.N. (2022). Evidence for Inhibitory Perturbations on the Amplitude, Gating, and Hysteresis of A-Type Potassium Current, Produced by Lacosamide, a Functionalized Amino Acid with Anticonvulsant Properties. Int. J. Mol. Sci..

[56] Liu P.Y., Chang W.T., Wu S.N. (2020). Characterization of the Synergistic Inhibition of I(K(erg)) and I(K(DR)) by Ribociclib, a Cyclin-Dependent Kinase 4/6 Inhibitor. Int. J. Mol. Sci..

[57] Cho H.Y., Chuang T.H., Wu S.N. (2021). Effective Perturbations on the Amplitude and Hysteresis of Erg-Mediated Potassium Current Caused by 1-Octylnonyl 8-[(2-hydroxyethyl)[6-oxo-6(undecyloxy)hexyl]amino]-octanoate (SM-102), a Cationic Lipid. Biomedicines.

[58] Yang C.S., Lai M.C., Liu P.Y., Lo Y.C., Huang C.W., Wu S.N. (2019). Characterization of the Inhibitory Effect of Gastrodigenin and Gastrodin on M-type K(+) Currents in Pituitary Cells and Hippocampal Neurons. Int. J. Mol. Sci..

[59] Albertson A.J., Bohannon A.S., Hablitz J.J. (2017). HCN Channel Modulation of Synaptic Integration in GABAergic Interneurons in Malformed Rat Neocortex. Front. Cell. Neurosci..

[60] Datunashvili M., Chaudhary R., Zobeiri M., Lüttjohann A., Mergia E., Baumann A., Balfanz S., Budde B., van Luijtelaar G., Pape H.C. (2018). Modulation of Hyperpolarization-Activated Inward Current and Thalamic Activity Modes by Different Cyclic Nucleotides. Front. Cell. Neurosci..

[61] Mellon D. (2016). Electrophysiological Evidence for Intrinsic Pacemaker Currents in Crayfish Parasol Cells. PLoS ONE.

[62] Chang W.T., Ragazzi E., Liu P.Y., Wu S.N. (2020). Effective block by pirfenidone, an antifibrotic pyridone compound (5-methyl-1-phenylpyridin-2[H-1]-one), on hyperpolarization-activated cation current: An additional but distinctive target. Eur. J. Pharmacol..

[63] Fürst O., D’Avanzo N. (2015). Isoform dependent regulation of human HCN channels by cholesterol. Sci. Rep..

[64] Elinder F., Männikkö R., Pandey S., Larsson H.P. (2006). Mode shifts in the voltage gating of the mouse and human HCN2 and HCN4 channels. J. Physiol..

[65] Rappaport S.M., Teijido O., Hoogerheide D.P., Rostovtseva T.K., Berezhkovskii A.M., Bezrukov S.M. (2015). Conductance hysteresis in the voltage-dependent anion channel. Eur. Biophys. J..

[66] Duret G., Polali S., Anderson E.D., Bell A.M., Tzouanas C.N., Avants B.W., Robinson J.T. (2019). Magnetic Entropy as a Proposed Gating Mechanism for Magnetogenetic Ion Channels. Biophys. J..

[67] Calejo A.I., Reverendo M., Silva V.S., Pereira P.M., Santos M.A., Zorec R., Gonçalves P.P. (2014). Differences in the expression pattern of HCN isoforms among mammalian tissues: Sources and implications. Mol. Biol. Rep..

[68] Bemme S., Weick M., Gollisch T. (2017). Differential Effects of HCN Channel Block on On and Off Pathways in the Retina as a Potential Cause for Medication-Induced Phosphene Perception. Investig. Opthalmol. Vis. Sci..

[69] Della Santina L., Bouly M., Asta A., Demontis G.C., Cervetto L., Gargini C. (2010). Effect of HCN channel inhibition on retinal morphology and function in normal and dystrophic rodents. Investig. Opthalmol. Vis. Sci..

[70] Popova E., Kupenova P. (2020). Effects of HCN channel blockade on the intensity-response function of electroretinographic ON and OFF responses in dark adapted frogs. Acta Neurobiol. Exp..

[71] Mares J. (2016). Lutein and Zeaxanthin Isomers in Eye Health and Disease. Annu. Rev. Nutr..

[72] Buscemi S., Corleo D., Di Pace F., Petroni M.L., Satriano A., Marchesini G. (2018). The Effect of Lutein on Eye and Extra-Eye Health. Nutrients.

[73] Fitzpatrick N., Chachay V., Bowtell J., Jackman S., Capra S., Shore A., Briskey D. (2022). An appraisal of trials investigating the effects on macular pigment optical density of lutein and zeaxanthin dietary interventions: A narrative review. Nutr. Rev..

[74] Mrowicka M., Mrowicki J., Kucharska E., Majsterek I. (2022). Lutein and Zeaxanthin and Their Roles in Age-Related Macular Degeneration-Neurodegenerative Disease. Nutrients.

[75] Thürmann P.A., Schalch W., Aebischer J.C., Tenter U., Cohn W. (2005). Plasma kinetics of lutein, zeaxanthin, and 3-dehydro-lutein after multiple oral doses of a lutein supplement. Am. J. Clin. Nutr..

[76] Bhat I., Mamatha B.S. (2021). Genetic factors involved in modulating lutein bioavailability. Nutr. Res..

[77] James Z.M., Borst A.J., Haitin Y., Frenz B., DiMaio F., Zagotta W.N., Veesler D. (2017). CryoEM structure of a prokaryotic cyclic nucleotide-gated ion channel. Proc. Natl. Acad. Sci. USA.

